# Preoperative echocardiography predictive analytics for postinduction hypotension prediction

**DOI:** 10.1371/journal.pone.0278140

**Published:** 2022-11-28

**Authors:** Manabu Yoshimura, Hiroko Shiramoto, Mami Koga, Yasuhiro Morimoto

**Affiliations:** Department of Anesthesiology, Ube Industries Central Hospital, Ube, Yamaguchi, Japan; Vita Salute University of Milan, ITALY

## Abstract

**Purpose:**

Hypotension is a risk factor for adverse perioperative outcomes. Preoperative transthoracic echocardiography has been extended for preoperative risk assessment before noncardiac surgery. This study aimed to develop a machine learning model to predict postinduction hypotension risk using preoperative echocardiographic data and compared it with conventional statistic models. We also aimed to identify preoperative echocardiographic factors that cause postinduction hypotension.

**Methods:**

In this retrospective observational study, we extracted data from electronic health records of patients aged >18 years who underwent general anesthesia at a single tertiary care center between April 2014 and September 2019. Multiple supervised machine learning classification techniques were used, with postinduction hypotension (mean arterial pressure <55 mmHg from intubation to the start of the procedure) as the primary outcome and 95 transthoracic echocardiography measurements as factors influencing the primary outcome. Based on the mean cross-validation performance, we used 10-fold cross-validation with the training set (70%) to select the optimal hyperparameters and architecture, assessed ten times using a separate test set (30%).

**Results:**

Of 1,956 patients, 670 (34%) had postinduction hypotension. The area under the receiver operating characteristic curve using the deep neural network was 0.72 (95% confidence interval (CI) = 0.67–0.76), gradient boosting machine was 0.54 (95% CI = 0.51–0.59), linear discriminant analysis was 0.56 (95% CI = 0.51–0.61), and logistic regression was 0.56 (95% CI = 0.51–0.61). Variables of high importance included the ascending aorta diameter, transmitral flow A wave, heart rate, pulmonary venous flow S wave, tricuspid regurgitation pressure gradient, inferior vena cava expiratory diameter, fractional shortening, left ventricular mass index, and end-systolic volume.

**Conclusion:**

We have created developing models that can predict postinduction hypotension using preoperative echocardiographic data, thereby demonstrating the feasibility of using machine learning models of preoperative echocardiographic data for produce higher accuracy than the conventional model.

## Introduction

Hypotension is an independent risk factor for adverse perioperative outcomes [1–4]. The early recognition of intraoperative hypotension may lead to preventive measures for improving anesthetic and surgical outcomes.

Currently, postinduction hypotension can be clinically predicted using a few available methods [5]. Various factors, including patient comorbidities, home medications taken on the day of surgery, and medications used to induce anesthesia, may precipitate postinduction hypotension.

Preoperative echocardiography is performed according to guidelines [6], which have been increasing in recent years [7]. When preoperative echocardiography results contain abnormalities, the findings must be interpreted to determine their clinical relevance. If patients do not have an active cardiac condition, those at risk for major adverse cardiac events before noncardiac surgery must still be identified.

Much data for developing robust predictive analytics can be processed using machine learning methods, eliminating many of the pitfalls and restrictions of standard modeling techniques [8–10]. Modern electronic health records combined with anesthesiology intraoperative records provide opportunities for complex clinical decision support tools that complement clinical training and experience when making decisions based on objective evidence and data.

In this study, we aimed to develop a machine learning model for the prediction of postinduction hypotension using information readily available from transthoracic echocardiography data, thereby demonstrating the viability of machine learning methods for intraoperative predictive analytics. We hypothesized that preoperative echocardiographic data help in predicting the risk of postinduction hypotension using a machine learning model rather than the conventional statistic models. We also aimed to identify preoperative echocardiographic factors that cause postinduction hypotension.

## Methods

The Institutional Review Board of Ube Industries Central Hospital, Ube City, Japan (2019–1103), approved the registry construction in November 2019. The Institutional Review Board waived the need for written informed patient consent. This article follows the STrengthening the Reporting of OBservational Studies in Epidemiology (STROBE) consensus guidelines and the "Guidelines for Developing and Reporting Machine Learning Predictive Models in Biomedical Research [11]." We obtained retrospective data for training and testing the machine learning algorithm from the electronic medical records. No direct patient identifiers were included in the data; there were no direct interactions with human subjects.

### Patient population

Noncardiac and non-obstetric surgery patients (aged >18 years) undergoing general anesthesia at the tertiary teaching hospital Ube Industries Central Hospital (Ube City, Japan) between April 2014 and September 2019 were included. We excluded patients who had already have been intubated and were using continuous catecholamines preoperatively. For patients who had undergone multiple procedures, we used data from their first procedure at this institution. We excluded patients who had not undergone preoperative echocardiography within one preoperative month. We also excluded patients who underwent echocardiography other than for preoperative purposes. The indications for preoperative echocardiography followed the American Heart Association preoperative guidelines [6].

### Data source

The perioperative electronic medical records used at Ube Industries Central Hospital were used to obtain all preoperative and intraoperative data. We retrieved the hospital’s electronic medical records using Data Warehouse with Fujitsu’s EGMAIN-EX to extract data. The electronic anesthesia chart was created by Nihon Koden Corporation and is PRIME Gaia. An engineer retrieved the echocardiographic data from data stored in the laboratory. All these data were linked and processed using a table. Data were aggregated from the electronic health records and made available by unified reports created by the hospital information technology department for reporting and research purposes.

### Data elements

Transthoracic echocardiography measurements were extracted from the demographic data; all measurements taken by preoperative echocardiography were included (S1 and S2 Tables). The measurements included recording state, arrhythmia, and heart rate. The intraoperative vital sign recorded the lowest blood pressure between post-intubation and the start of surgery in eligible patients, associated with echocardiographic data.

### Primary outcome

Postinduction hypotension was the primary outcome. It was defined as any single mean arterial pressure (MAP) of <55 mmHg on any non-invasive or continuous blood pressure measurement within the start of surgery recorded by the induction time of general anesthesia. Achievement of hypotension was limited only to the available recorded data. Any recorded blood pressure measurement was included, whether from an arterial line or a non-invasive blood pressure cuff. Blood pressures taken by a non-invasive blood pressure cuff were recorded; therefore, the frequency depended on the cycle time designated by the anesthesiologist. Continuous blood pressure measurements were recorded in 1-min cycles.

### Feature selection

Selecting the appropriate set of features is critical for high model accuracy and interpretability. We used the SHapley Additive exPlanations (SHAP) method [12], which assigns a value to each feature for each prediction by calculating a weighted average of differences between models with one feature included and withheld for all possible features subsets. We could calculate the Shapley value based on the magnitude of the feature attributions, which indicates how much each feature contributes to the target variable in our machine learning model. With a higher SHAP value, the attribution of this feature to predicting the risk becomes larger. Therefore, the SHAP values can be used to explain the output of our model.

### Model selection

**Fig 1:**
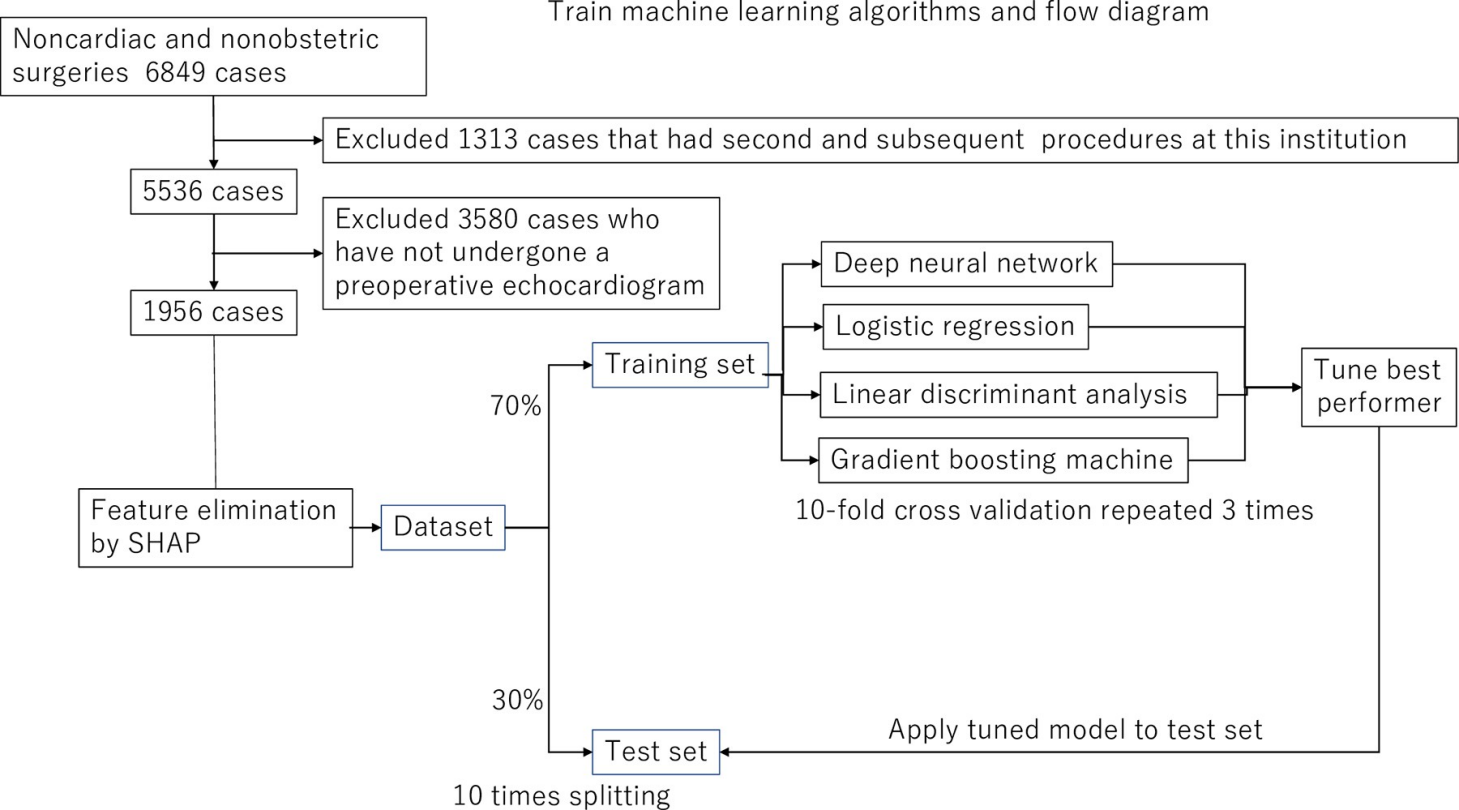
Diagram of methods. The complete data set was split into training and test sets ten times. The machine learning methods were trained, and the best performer was selected for additional parameter tuning before being applied to the test set for validation.

Data were randomly separated into training (70%) and test (30%) sets for validation (Fig 1). We utilized random numbers to produce the test data 10 times. Specifically, 70% of the data were used for training the machine-learned models, and 30% were used for the test set. Ten-fold cross-validation was used to train machine learning algorithms on the training set in no predetermined order. Variable selection and all tuning aspects were repeated within each of the 10-fold cross-validations. The area under the receiver operating characteristic curve (AUROC) was used because of concerns regarding how various machine learning models treat class imbalances. We represent the AUROC and the specificity, sensitivity, positive predictive value, and negative predictive value of each machine learning model to predict the optimal cut-off postinduction hypotension. The optimal cut-off belongs to the threshold at which specificity + sensitivity − 1 is maximized.

The following algorithms were trained: logistic regression (LR), linear discriminant analysis (LDA), gradient boosting machine (XGB), and deep neural network (DNN). Although the goal was to develop a predictive model, an additional aim was to explore how various machine learning algorithms compare concerning handling preoperative transthoracic echocardiography and intraoperative data.

The "caret" package in R was utilized for the initial training and 10-fold cross-validation, using the ROC as the performance metric and basic tuning on parameters specific to each method of machine learning. Tuning was produced by a narrow grid search as dictated by the defaults in the package [13]. The library "keras" used for DNNs was challenging to handle in caret; therefore, we adjusted them individually.

Tuning parameters were estimated by an expanded default grid search wherein extensive but realistic ranges of values are given for each tuning parameter, and the performance of the resulting models was compared.

Because not all machine learning algorithms comply with variable importance calculations, we determined the variable importance for the final model of gradient boosting. Variable importance is calculated based on how valuable any given feature is to support the classification process when the classifier is built. Its effect on the performance measure determines it. Generally, variable importance helps evaluate the impact of any given variable on the performance of the algorithm. The performance declines if a variable with high importance is permuted or removed from the model. The greater the importance, the more necessary the variable is to the performance of the model.

The algorithm’s generalizability and assessing whether the model was overfitted were determined by simulating the final model on the test set. Fig 1 depicts the process. The decision curve analysis incorporates the information about the benefit of correct hypotension (true-positive) and the relative harm of the over triage (false-positive; i.e., the net benefit) over a threshold range of probabilities of the outcome. We used R software (R Foundation for Statistical Computing version 3.5, Austria) to perform all statistical operations.

### Missing data

There were no missing blood pressure data. Missing data accounted for 44% of echocardiography data. However, we did not consider missing echocardiographic data to be simply missing data. Echocardiography does not take detailed measurements of what appears to be normal. For example, if the aortic valve is firmly open, then a continuous formula or planimetry will not identify the valve opening area. Moreover, if mitral regurgitation is not observed, then vena contracta will not be measured. We substituted the missing data with normal values [14] because this is characteristic of the echocardiographic examination.

### Sensitivity analysis

Hypotension was defined as MAP <55 mmHg due to its connection with particular surgical outcomes. Other studies support a more conservative definition of hypotension, namely that MAP <65 mmHg is related with injuries such as renal damage [15]. Therefore, we conducted a sensitivity study in which hypotension was defined as MAP <65 mmHg, and we trained the algorithm with the greatest performance on this revised definition to develop a new model.

## Results

**Fig 2:**
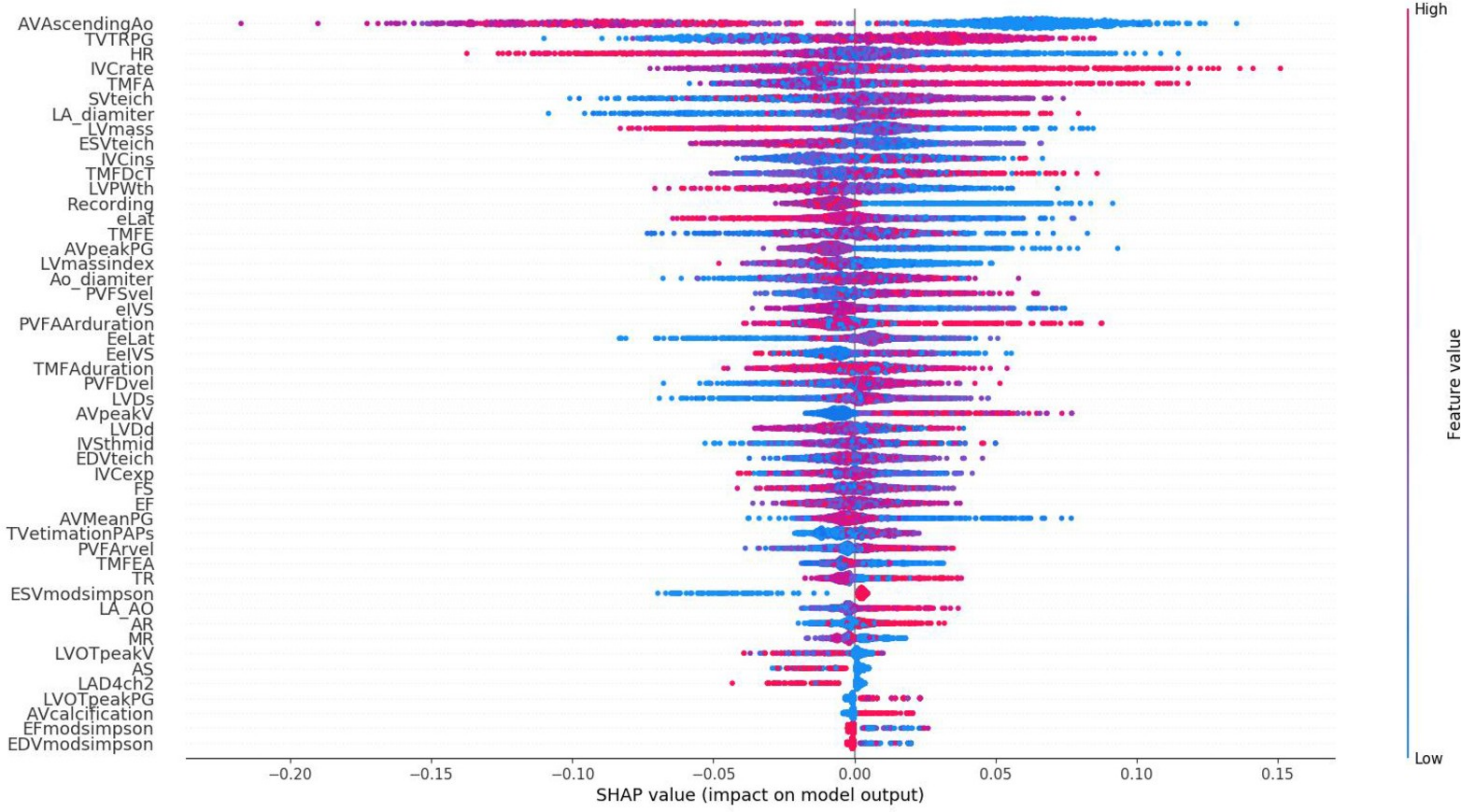
Reduction of dimensionality by the SHapley Additive exPlanations (SHAP) method. The number of features used for training was reduced from 95 to 49. Each point in the figure is a Shapley value of a feature. The x-axis shows the Shapley value, and the y-axis shows the feature importance. Each value of each variable is colored by value, and its position on the x-axis is shown as a single dot indicating the impact that value of the variable had on the model prediction in logit space. Variables with a wide spread have a greater impact on model predictions. The colors indicate whether low or high values of each variable affect risk and in which direction. AVAscendingAo, ascending aorta diameter; TVTRPG, tricuspid regurgitation pressure gradient; HR, heart rate; IVCrate, inferior vena cava inspiratory/expiratory rate; TMFA, transmitral flow A wave; SVteich, stroke volume (Teichholz); LA_diamiter, left atrium diameter; LVmass, left ventricular mass; ESVteich, end systolic volume (Teichholz); IVCins, inferior vena cava inspiratory diameter; TMFDcT, transmitral flow deceleration time; LVPWth, thickness of the left ventricular posterior wall; Recording, recording state; eLat, e wave lateral; TMFE, transmitral flow E wave; AVpeakPG, aortic valve peak pressure gradient; LVmaindex, left ventricular mass index; Ao_diamiter, Aortic diameter; PVFSvel, pulmonary venous flow S wave velocity; eIVS, e wave interventricular septum; PVFAArduration, pulmonary venous flow Ar duration; EeLat, lateral E/e ratio; EeIVS, interventricular E/e ratio; TMFAduration, transmitral flow A duration; PVFDvel, pulmonary venous flow D wave velocity; LVDs, left ventricular diameter at the end systole; AVpeakV, aortic valve peak velocity; LVDd, left ventricular diameter at the end diastole; IVSthmid, thickness of the interventricular septum mid wall; EDVteich, end diastolic volume (Teichholz); IVCexp, inferior vena cava expiratory diameter; FS, fractional shortening; EF, ejection fraction; AVMeanPG, aortic valve mean pressure gradient; TVestimationPAPs, pulmonary arterial pressure; PVFArvel, pulmonary venous flow Ar velocity; TMFEA, transmitral flow E/A ratio; TR, tricuspid regurgitation; ESVmodsimpson, end systolic volume (modified Simpson’s method); LA_AO, left atrium/aortic ratio; AR, aortic regurgitation; MR, mitral regurgitation; LVOTpeakV, left ventricular outflow tract peak velocity; AS, aortic stenosis; LAD4ch2, left atrium diameter from 4 chamber; LVOTpeakPG, left ventricular outflow tract peak pressure gradient; AVcalcification, aortic valve calcification; EFmodsimpson, ejection fraction (modified Simpson’s method); EDVmodsimpson, end diastolic volume (modified Simpson’s method).

This study included 6,849 patients undergoing noncardiac and non-obstetric surgery cases (aged >18 years) receiving general anesthesia without using continuous catecholamines and intubation before surgery. We found no cases of extreme fluid loading (>80 ml/hr) or prophylactic use of vasopressor and vasodilator before induction. In total, 1,313 cases had undergone multiple procedures; we used the data from their first procedure at this institution. We excluded 3,580 patients who had not undergone preoperative echocardiography within one preoperative month, who had echocardiography other than preoperative purposes, or had no echocardiography examination. Finally, we included 1,956 cases. S2 Table details the data characteristics of the complete data set. Of the remaining cases, 670 (34%) experienced postinduction hypotension. In total, the training set contained 1,369 cases, and the test set contained 587 cases. Using SHAP, the number of features was reduced from 95 to 49 (Fig 2). The 49 features selected using SHAP were used in the all model.

**Fig 3:**
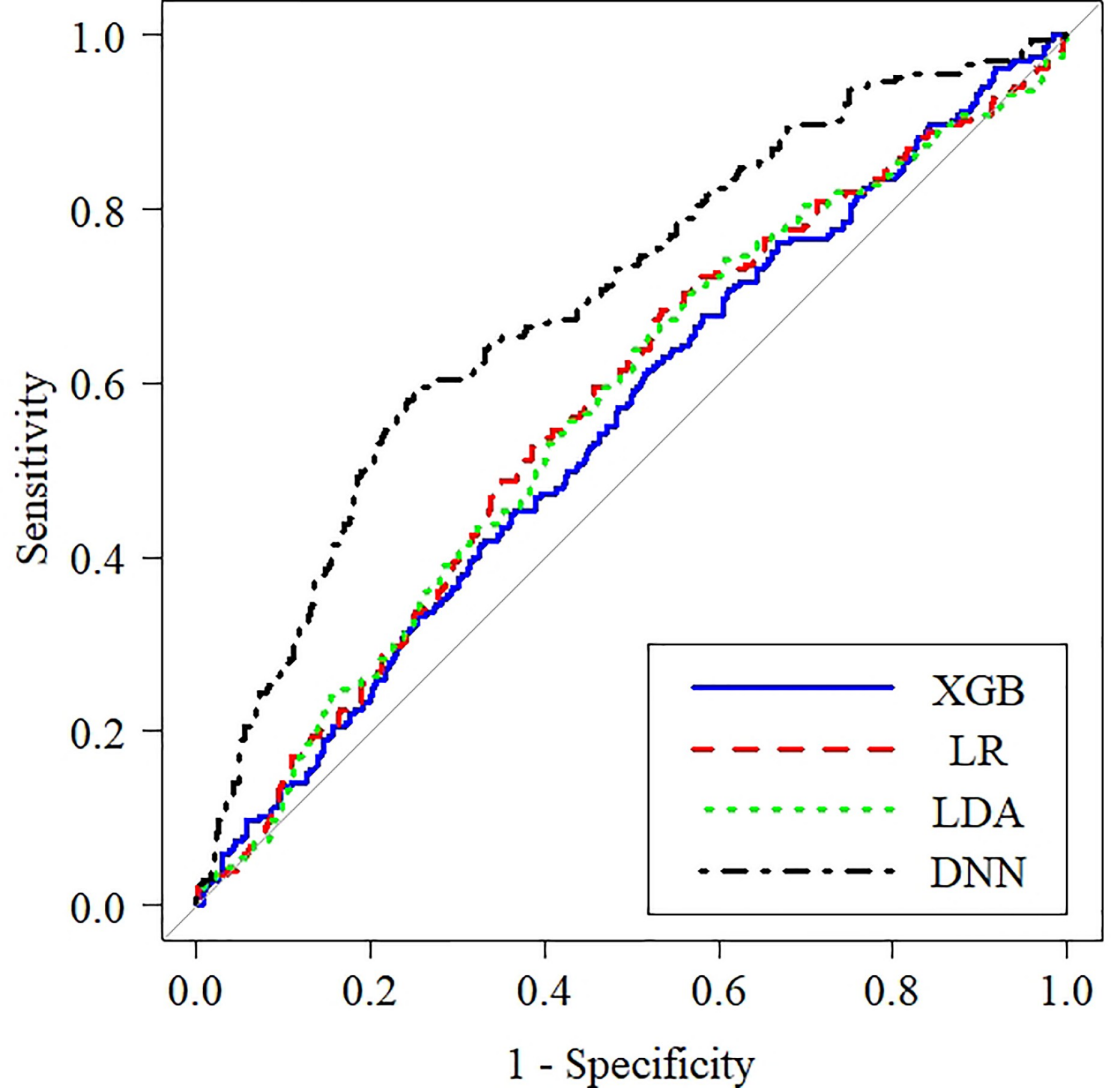
Receiver operating characteristic curves of machine learning methods for predicting postinduction hypotension in the test data set. The greater the area under the receiver operating characteristic curve, the higher the discriminative ability of the model. LR; logistic regression, LDA; linear discriminant analysis, XGB; gradient boosting machine, DNN; deep neural network.

**Fig 4:**
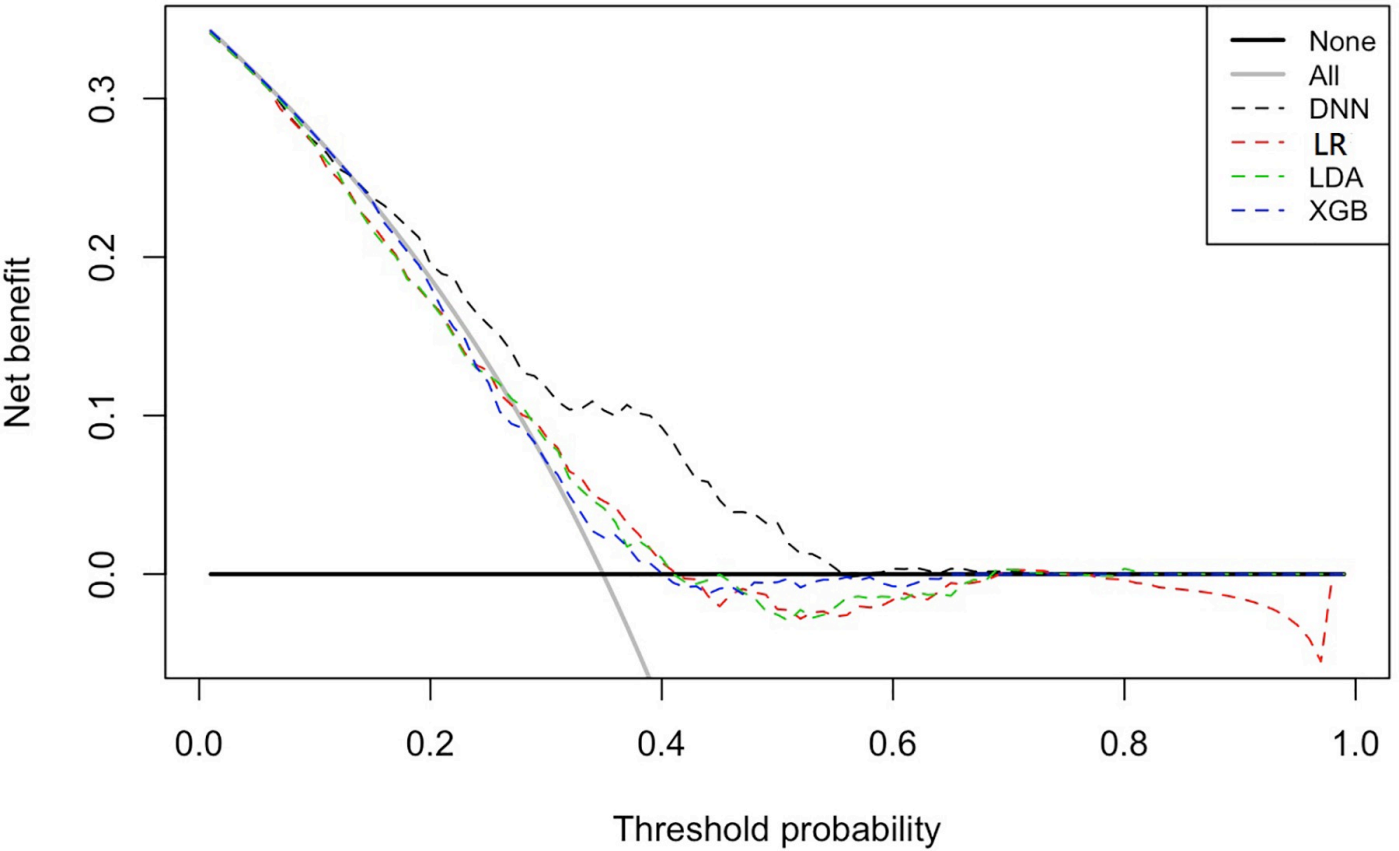
Decision curve analysis. Decision curve analysis allows the variation of threshold probability to examine whether one model is superior to another at a specific range of threshold probability, for the net benefit. This figure indicates that DNN is superior when the threshold is 0.2–0.55. LR; logistic regression, LDA; linear discriminant analysis, XGB; gradient boosting machine, DNN; deep neural network.

**Fig 5:**
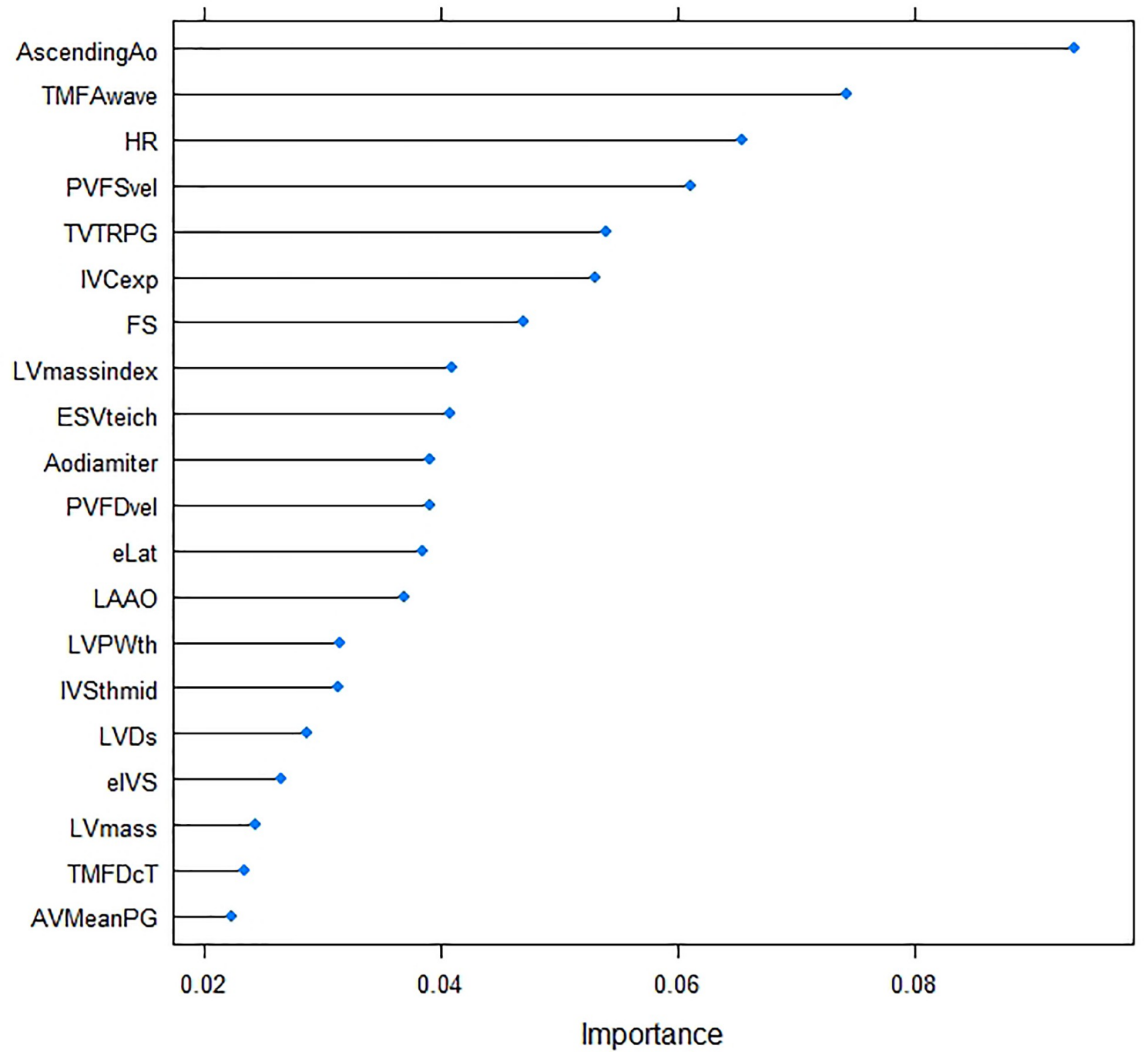
Variable importance of features included in the gradient boosting machine learning (XGB) algorithm for predicting postinduction hypotension. Variable importance is computed according to the degree of importance of any given feature to aid during classification when the classifier is built, determined by its effect on the performance measure. The greater the importance, the more essential the variable is to the performance of the model. Ao; aorta, TMF; trans mitral flow, HR; heart rate, PVF; pulmonary venous flow, TVTRPG; tricuspid regurgitation pressure gradient, IVC; inferior vena cava, FS; fractional shortening, LV; left ventricular, ESV; end-systolic volume, LA; left atrium, LVPWth; thickness of the left ventricular posterior wall, IVS; interventricular septum, LVDd; left ventricular diameter at the end diastole, AV; aortic valve, PG; pressure gradient.

After training, the AUROC run on the test set using DNN was 0.72 (95% confidence interval (CI) = 0.67–0.76), XGB was 0.54 (95% CI = 0.51–0.59), LDA was 0.56 (95% CI = 0.51–0.61), and LR was 0.56 (95% CI = 0.51–0.61); positive predictive values of DNN were 0.86 (95% CI = 0.81–0.90); and negative predictive values of DNN were 0.49 (95% CI = 0.43–0.55), as shown in Table 1 and Fig 3. Fig 4 shows each model’s net benefit through a range of threshold probabilities of the outcome as a decision curve. Fig 5 shows the variable importance based on XGB. Variables of high importance include the ascending aorta diameter, transmitral flow A wave, heart rate, pulmonary venous flow S wave, tricuspid regurgitation pressure gradient (TVTRPG), inferior vena cava expiratory diameter (IVC exp), fractional shortening (FS), left ventricular (LV) mass index, and end-systolic volume (ESV) at Fig 5.

**Table 1:** Area under the receiver operating characteristic curves (AUROC) for each machine learning classifier on the test data set.

Model	AUC (95% CI)	Sensitivity (95% CI)	Specificity (95% CI)	PPV (95% CI)	NPV (95% CI)
Linear discriminant analysis	0.56	0.68	0.45	0.40	0.73
(LDA)	(0.51–0.61)	(0.61–0.75)	(0.40–0.50)	(0.35–0.46)	(0.67–0.78)
Logistic regression	0.56	0.66	0.46	0.41	0.73
(LR)	(0.51–0.61)	(0.59–0.73)	(0.41–0.51)	(0.35–0.46)	(0.67–0.79)
Gradient boosting machine	0.54	0.66	0.44	0.38	0.70
(XGB)	(0.51–0.59)	(0.60–0.73)	(0.39–0.49)	(0.33–0.44)	(0.64–0.76)
Deep neural network	0.72	0.65	0.70	0.86	0.49
(DNN)	(0.67–0.76)	(0.60–0.70)	(0.64–0.76)	(0.81–0.90)	(0.43–0.55)

### Sensitivity analysis

The DNN model with a different definition of hypotension than the primary analysis (MAP < 65 mmHg) had an AUROC of 0.56 (95% CI = 0.53–0.59), with a specificity of 64% and sensitivity of 64%. Furthermore, the AUROCs of XGB, LDA, and LR were 0.51 (95% CI = 0.47–0.55), 0.52 (95% CI = 0.48–0.56), and 0.52 (95% CI = 0.48–0.56), respectively.

## Discussion

We examined the use of machine learning methods—based on existing information in the preoperative transthoracic echocardiography measurements—for intraoperative predictive analytics, specifically for predicting postinduction hypotension. DNN performed better than XBG, LDA, and LR. Variables of high importance include the ascending aorta diameter, transmitral flow A wave, heart rate, pulmonary venous flow S wave, TVTRPG, IVC exp, FS, LV mass index, and ESV. The sensitivity analysis generally showed the same direction of results, indicating the robustness of the results.

There are numerous benefits of using machine learning for problems. The most obvious of these is incorporating a large amount of disparate data into a unified algorithm. Most machine learning methods are highly scalable and thus can handle various problems with different features. When the limits of human understanding have been superseded, machine learning can be particularly useful [8, 9, 16–18]. For example, despite a thorough understanding of pharmacology, normal physiology [19], pathophysiology, and surgical factors [18, 20], postinduction hypotension [21] still occurs at a surprisingly high rate, possibly because the number of variables involved is so vast and complex. Thus, such a problem is a prime target for machine learning.

Some machine learning methods offer information regarding various features, such as the variable importance shown for the gradient boosting machine algorithm. Machine learning tools tend to effectively be "black boxes." Initially, we attempted to solve this problem to some extent by using SHAP. Lundberg and Lee [12] proposed SHAP for better interpretability of machine learning models instead of a black box model. This point is significantly crucial in medicine. If we can determine which features explain the target variable better than others, we can exploit those features for better treatment.

We believed that the principle of using SHAP to reduce the number of features could be used for DNNs, which require a large sample size. Because DNNs allow the user to control the complexity of the model based on the number of data (number of layers, number of nodes, model regularization, etc.), even when the number of data is small, as was the case here, the DNN could produce a reasonable area under the curve (AUC) by selecting the most appropriate parameters and reducing the number of features. Moreover, because the data in this study comprised such a system that a relatively small DNN model was able to reproduce the results, it is thought that the DNN was able to produce a relatively high AUC.

In our study, we used MAP <55 mmHg as the criterion for postinduction hypotension. The criteria for intraoperative hypotension remain undefined [22]. In this study, we adopted strict criteria. We believe that this will allow us to more clearly identify those with hypotension. Robustness was complemented by performing a sensitivity analysis with MAP <65 mmHg.

Two studies have investigated the relationship between preoperative echocardiography and postinduction hypotension. Szabo et al. reported the relationship between IVC and intraoperative hypotension [23], Fiza et al. also conducted a prospective study on preoperative echocardiography and postinduction hypotension [24], but found no significant association between hypotension and IVC, left ventricular diastolic dimension. They found no significant association between IVC and left ventricular diastolic dimension, possibly due to the small sample size. In addition, they performed point-of-care echocardiography, not formal echocardiography. Our study identified IVC as an essential factor that affects predictive models. Other variables of importance also make sense when you consider that hypotension is associated with age and a history of hypertension because the ascending aorta diameter, transmitral flow A wave, heart rate, pulmonary venous flow S wave, TVTRPG, IVC exp, FS, LV mass index, and ESV are related to age and a history of hypertension. This finding explains that the results of variable importance may have been used as proxy points for age and hypertension. The process of selecting variable importance is unknown, but the results are reasonable. Cheung et al. used a logistic regression model to search for predictors of intraoperative hypotension [25], consistent with their results that our DNN model also reflects age and hypertension.

AUC did not show excellent values in our study. We aimed to create a model that fits the preoperative echocardiographic data. Therefore, only the default grid search was used, but the details of each model’s parameters were unchanged. If the parameters are adjusted in detail, it may be possible to calculate better AUC for models other than DNN. We could not follow the low NPV value of DNN, we could not follow it because the model was constructed with an emphasis on AUC. Nevertheless, it is a useful model for identifying postinduction hypotension because of its high specificity and PPV.

Some meta-analyses have suggested that machine learning is inferior to LR [26], but meta-analyses suggest that machine learning is superior to LR in the cardiovascular field [27]. Ultimately, it appears that the only way to find the model that best fits the nature of the data is to search for the model that best fits the nature of the data, as in this case.

This study has some limitations. First, this was a single-center retrospective study. Therefore, selection bias is possible. However, to the best of our knowledge, no published study has evaluated the intraoperative period using all echocardiographic data. Second, there are numerous missing data; however, because of the nature of echocardiography, measuring all the parameters does not make clinical sense. We were able to utilize all echocardiographic data because we substituted the missing data with normal values. Third, the anesthetic drug used for the induction and the patient’s background was not considered in predicting hypotension. However, we reviewed all of the cases and found no cases of extreme fluid loading or prophylactic use of vasopressor and vasodilator before induction. Whenever possible, we removed biases that can cause postinduction hypotension. We also identified preoperative echocardiographic factors that can cause postinduction hypotension.

We have created developing models that can predict postinduction hypotension using preoperative echocardiographic data, thereby demonstrating the feasibility of using machine learning models of preoperative echocardiographic data for produce higher accuracy than the conventional model.

## Supporting information

S1 Table Background of patient who received preoperative echocardiogram. (DOCX)

S2 Table Transthoracic echocardiography measurement. (DOCX)
