# Supplementary material for: Preoperative echocardiography predictive analytics for postinduction hypotension prediction
Source: PLoS One. 2022 Nov 28;17(11):e0278140. doi: 10.1371/journal.pone.0278140 (PMC9704611; doi:10.1371/journal.pone.0278140)
Supplement: S1 Table — (DOCX) [file pone.0278140.s001.docx]

| Factor | Group | No hypotension | Hypotension | p.value |
| --- | --- | --- | --- | --- |
| n |  | 1287 | 670 |  |
| age (years) | | 78.0 [69.0, 84.0] | 78.0 [71.0, 86.0] | 0.004 |
| length (cm) | | 156.0 [150.0, 164.0] | 154.0 [147.0, 161.0] | <0.001 |
| weight (kg) | | 53.9 [45.5, 63.6] | 52.7 [45.0, 60.0] | 0.008 |
| surgical type (%) | General gurgery | 456 (35.4) | 209 (31.2) | NA |
|  | Neurosurgery | 120 ( 9.3) | 92 (13.7) | |
|  | Orthopedic surgery | 617 (48.0) | 326 (48.7) | |
|  | Urology | 14 ( 1.1) | 6 ( 0.9) |  |
|  | Dermatology | 54 (4.3) | 23 (3.4) |  |
|  | Ophthalmology | 17 (1.3) | 10 (1.4) |  |
|  | others | 9 (0.7) | 4 (0.4) |  |
| ASA (%) | 1 | 86 ( 6.7) | 30 ( 4.5) | NA |
|  | 2 | 750 (58.3) | 406 (60.6) | |
|  | 3 | 338 (26.3) | 185 (27.6) | |
|  | 4 | 1 ( 0.1) | 0 ( 0.0) |  |
|  | 1E | 5 ( 0.4) | 0 ( 0.0) |  |
|  | 2E | 50 ( 3.9) | 15 ( 2.2) |  |
|  | 3E | 55 ( 4.3) | 32 ( 4.8) |  |
|  | 4E | 2 ( 0.2) | 2 ( 0.3) |  |
|  |  |  |  |  |
| gender (%) | female | 694 (53.9) | 421 (62.8) | <0.001 |
|  | male | 593 (46.1) | 249 (37.2) | |
| mean blood pressure on admission (mmHg) | | 103.0 [93.3, 113.0] | 99.00 [88.7, 109.3] | <0.001 |
| heart rate on admission (bpm) | | 75.0 [65.0, 85.0] | 72.00 [65.0, 84.0] | 0.031 |
|  |  |  |  |  |
| (median [IQR]) | |  |  |  |

S1 Table. Background of patient who received preoperative echocardiogram
