# Supplementary material for: Preoperative echocardiography predictive analytics for postinduction hypotension prediction
Source: PLoS One. 2022 Nov 28;17(11):e0278140. doi: 10.1371/journal.pone.0278140 (PMC9704611; doi:10.1371/journal.pone.0278140)
Supplement: S2 Table — (DOCX) [file pone.0278140.s002.docx]

| TTE measurement | Group | No hypotension | hypotension | p.value |
| --- | --- | --- | --- | --- |
| n |  | 1286 | 670 |  |
| Recording (%) | poor | 240 (18.7) | 159 (23.7) | 0.006 |
|  | fair | 747 (58.1) | 387 (57.8) |  |
|  | good | 299 (23.3) | 124 (18.5) |  |
| rhythm (%) | arrythmia | 177 (13.8) | 83 (12.4) | 0.44 |
|  | sinus rhythm | 1109 (86.2) | 587 (87.6) |  |
| HR (bpm) |  | 75 [38, 162] | 72 [47, 124] | 0.033 |
| Ao_diamiter (mm) |  | 32.0 [18.7, 46.0] | 32.0 [21.0, 48.0] | 0.264 |
| LA_diamiter (mm) |  | 36.0 [15.0, 74.1] | 36.2 [19.0, 61.1] | 0.288 |
| LA/Ao |  | 1.1 [0.5, 2.5] | 1.1 [0.6, 2.0] | 0.311 |
| IVSth_base (mm) |  | 10.0 [3.0, 20.0] | 10.00 [1.0, 17.0] | 0.178 |
| IVSth_mid (mm) |  | 9.3 [4.1, 21.3] | 9.2 [5.1, 19.7] | 0.775 |
| LVPWth (mm) |  | 9.2 [3.8, 17.4] | 9.1 [4.6, 14.0] | 0.189 |
| LVDd (mm) |  | 45.0 [20.5, 69.0] | 44.5 [28.2, 67.3] | 0.098 |
| LVDs (mm) |  | 28.4 [5.0, 58.9] | 28.3 [12.0, 57.0] | 0.414 |
| FS (%) |  | 36.3 [7.9, 79.5] | 36.5 [4.4, 59.0] | 0.749 |
| EDV_teich (ml) |  | 93.2 [13.4, 247.1] | 91.0 [30.0, 233.7] | 0.163 |
| ESV_teich (ml) |  | 31.0 [10.2, 172.7] | 30.6 [4.2, 159.7] | 0.496 |
| SV_teich (ml) |  | 60.7 [12.0, 129.3] | 58.9 [7.9, 142.5] | 0.113 |
| EF (%) |  | 65.9 [17.6, 90.8] | 66.1 [10.1, 89.1] | 0.725 |
| EDV_mod_simpson (ml) |  | 115.0 [25.5, 246.8] | 115.0 [15.1, 209.9] | 0.223 |
| ESV_mod_simpson (ml) |  | 61.0 [7.0, 162.3] | 61.0 [5.0, 128.4] | 0.863 |
| SV_mod_simpson (ml) |  | 70.0 [10.3, 130.5] | 70.0 [10.2, 83.4] | 0.667 |
| EF_mod_simpson (%) |  | 60.0 [25.6, 86.0] | 60.0 [23.2, 82.2] | 0.537 |
| TMF_Ewave (cm/s) |  | 66.4 [0.4, 174.0] | 66.2 [28.7, 336.0] | 0.971 |
| TMF_Awave (cm/s) |  | 82.0 [0.0, 195.3] | 84.6 [0.1, 180.7] | 0.002 |
| TMF_E/A (excluding arrithmia) |  | 0.70 [0.30, 4.0] | 0.70 [0.30, 4.0] | 0.047 |
| TMF_DcT (ms) |  | 240.0 [55.5, 777.4] | 240.0 [58.0, 771.8] | 0.357 |
| TMF_Awave_duration (ms) |  | 133.1 [0.0, 325.3] | 133.2 [0.0, 224.5] | 0.246 |
| PVF_Svel (cm/s) |  | 56.0 [25.8, 313.0] | 56.5 [18.8, 115.1] | 0.037 |
| PVF_Dvel (cm/s) |  | 45.3 [16.0, 107.2] | 44.5 [16.4, 93.6] | 0.967 |
| PVF_Arvel (cm/s) |  | 25.1 [13.5, 111.4] | 25.8 [16.2, 81.4] | 0.312 |
| PVF_Ar_duration (ms) |  | 96.1 [0.0, 213.0] | 99.8 [0.0, 199.6] | 0.243 |
| e_IVS (cm/s) |  | 7.0 [1.9, 18.1] | 7.0 [2.4, 67.0] | 0.014 |
| e_Lat (cm/s) |  | 10.0 [1.8, 27.0] | 10.0 [1.4, 18.0] | 0.059 |
| E/e_IVS |  | 8.0 [4.3, 34.9] | 8.0 [1.0, 28.0] | 0.144 |
| E/e_Lat |  | 8.0 [2.6, 37.1] | 8.0 [3.3, 65.0] | 0.426 |
| IVC_exp (mm) |  | 13.8 [3.0, 29.5] | 13.4 [5.8, 27.1] | 0.296 |
| IVC_ins (mm) |  | 4.5 [0.9, 25.4] | 4.3 [1.6, 22.0] | 0.049 |
| IVC_rate (%) |  | 65.7 [9.1, 91.0] | 66.7 [6.1, 100.0] | 0.068 |
| LV_mass (g) |  | 140.0 [15.0, 1055.6] | 137.5 [51.3, 334.0] | 0.07 |
| LV_mass_index (g/m2) |  | 81.3 [13.6, 632.1] | 77.4 [45.0, 184.1] | 0.097 |
| LVOT_peakPG (mmHg) |  | 3.0 [1.1, 39.5] | 3.0 [2.2, 14.2] | 0.142 |
| LVOT_peakV (m/s) |  | 0.9 [0.0, 3.1] | 0.9 [0.7, 1.9] | 0.189 |
| LVOT_SV (ml) |  | 70.0 [0.0, 134.6] | 70.0 [35.4, 154.4] | 0.651 |
| CO (l/min) |  | 6.00 [2.6, 15.8] | 6.0 [2.7, 26.5] | 0.1 |
| CI (l/min/m2) |  | 3.30 [1.9, 11.80] | 3.30 [1.9, 20.4] | 0.404 |
| LA_diamiter_4ch1 (mm) |  | 46.0 [33.0, 84.0] | 46.00 [31.0, 94.0] | 0.047 |
| LA_diamiter_4ch2 (mm) |  | 35.0 [28.0, 67.0] | 35.00 [33.0, 72.0] | 0.014 |
| LA_volume_index (ml/m2) |  | 22.0 [9.8, 91.5] | 22.0 [22.0, 90.9] | 0.369 |
| LA_volume (ml) |  | 37.0 [12.3, 157.0] | 37.0 [37.0, 166.4] | 0.209 |
| RA_diamiter_4ch1 (mm) |  | 34.0 [30.0, 82.0] | 34.0 [32.0, 78.0] | 0.003 |
| RA_diamiter_4ch2 (mm) |  | 34.0 [20.0, 68.0] | 34.0 [24.0, 59.0] | 0.303 |
| RVDd (mm) |  | 20.0 [20.0, 54.0] | 20.0 [20.0, 58.0] | 0.487 |
| RVWth (mm) |  | 5.0 [5.0, 8.0] | 5.0 [5.0, 40.0] | 0.237 |
| tamponade (%) | 0 | 1228 (95.5) | 643 (96.0) | 0.726 |
|  | 1 | 58 ( 4.5) | 27 ( 4.0) |  |
| tampo_LA_lat1 (mm) |  | 0.0 [0.0, 16.0] | 0.0 [0.0, 0.0] | 0.307 |
| tampo_LA_lat2 (mm) |  | 0.0 [0.0, 12.0] | 0.0 [0.0, 0.0] | 0.307 |
| tampo_LV_inf1 ( mm) |  | 0.0 [0.0, 18.0] | 0.0 [0.0, 18.0] | 0.815 |
| tampo_LV_inf2 (mm) |  | 0.0 [0.0, 22.0] | 0.0 [0.0, 23.0] | 0.997 |
| tampo_RA_lat1 (mm) |  | 0.0 [0.0, 36.0] | 0.0 [0.0, 20.0] | 0.673 |
| tampo_RA_lat2 (mm) |  | 0.0 [0.0, 23.0] | 0.0 [0.0, 21.0] | 0.675 |
| tampo_RV_ante1 (mm) |  | 0.0 [0.0, 25.0] | 0.0 [0.0, 13.0] | 0.592 |
| tampo_RV_ante2 (mm) |  | 0.0 [0.0, 41.0] | 0.0 [0.0, 22.0] | 0.672 |
| tampo_Collapse (%) | 0 | 1281 (99.6) | 666 (99.4) | 0.502 |
|  | 1 | 5 ( 0.4) | 4 ( 0.6) |  |
| AS (%) | normal | 1167 (90.8) | 611 (91.2) | 0.779 |
|  | trivial | 4 ( 0.3) | 0 ( 0.0) |  |
|  | mild | 66 ( 5.1) | 32 ( 4.8) |  |
|  | moderate | 33 ( 2.6) | 20 ( 3.0) |  |
|  | severe | 16 ( 1.2) | 7 ( 1.0) |  |
| AR (%) | normal | 769 (59.8) | 398 (59.4) | 0.823 |
|  | trivial | 448 (34.8) | 237 (35.4) |  |
|  | mild | 65 ( 5.1) | 33 ( 4.9) |  |
|  | moderate | 2 ( 0.2) | 1 ( 0.1) |  |
|  | severe | 2 ( 0.2) | 1 ( 0.1) |  |
| AV_calcification (%) | 0 | 1117 (86.9) | 586 (87.5) | 0.723 |
|  | 1 | 169 (13.1) | 84 (12.5) |  |
| AV_peakV (m/s) |  | 1.5 [0.1, 4.7] | 1.5 [0.0, 4.9] | 0.245 |
| AV_peakPG (mmHg) |  | 16.0 [2.0, 86.7] | 16.0 [2.4, 94.3] | 0.259 |
| AV_MeanPG (mmHg) |  | 10.0 [1.6, 53.3] | 10.0 [2.0, 46.8] | 0.166 |
| AVA_2D (cm2) |  | 3.0 [0.6, 3.8] | 3.0 [0.7, 3.0] | 0.654 |
| AVA_2D_BSA (cm2/m2) |  | 1.2 [0.4, 2.6] | 1.2 [0.5, 1.9] | 0.175 |
| AVA_continuous (cm2) |  | 3.0 [0.4, 3.0] | 3.0 [0.6, 3.0] | 0.564 |
| AVA_continuous_BSA (cm2/m2) |  | 1.2 [0.4, 1.6] | 1.2 [0.5, 2.2] | 0.034 |
| AR_PHT (msec) |  | 500.0 [251.0, 898.6] | 500.0 [230.0, 971.5] | 0.745 |
| AR_VC (mm) |  | 0.0 [0.0, 8.0] | 0.0 [0.0, 10.0] | 0.694 |
| AV_surgical_annulus (mm) |  | 21.0 [16, 41.0] | 21.0 [16.0, 26.8] | 0.484 |
| Valsalva (mm) |  | 29.0 [27.0, 48.0] | 29.0 [29.0, 48.0] | 0.486 |
| STJ (mm) |  | 25.0 [21.0, 40.0] | 25.00 [25.0, 42.0] | 0.643 |
| Ascending_Ao (mm) |  | 26.0 [16.1, 49.5] | 26.0 [24.0, 57.0] | 0.003 |
| MS (%) | normal | 1270 (98.7) | 665 (99.3) | 0.943 |
|  | trivial | 2 ( 0.2) | 0 ( 0.0) |  |
|  | mild | 9 ( 0.7) | 4 ( 0.6) |  |
|  | moderate | 4 ( 0.3) | 1 ( 0.1) |  |
|  | severe | 1 ( 0.1) | 0 ( 0.0) |  |
| MR (%) | normal | 486 (37.8) | 261 (38.9) | 0.491 |
|  | trivial | 238 (18.5) | 124 (18.5) |  |
|  | mild | 490 (38.1) | 242 (36.1) |  |
|  | moderate | 70 ( 5.5) | 39 ( 5.8) |  |
|  | severe | 2 ( 0.2) | 4 ( 0.6) |  |
| MV_state | normal | 1206 (93.8) | 615 (91.8) | 0.273 |
|  | calcification | 54 (4.2) | 41 (4.1) |  |
|  | prolapse | 24 (1.9) | 13 (1.9) |  |
|  | SAM | 2 (0.2) | 1 (0.1) |  |
| MV_peakV (m/s) |  | 5.0 [1.1, 5.0] | 5.0 [1.5, 5.0] | 0.595 |
| MV_MeanPG (mmHg) |  | 3.0 [2.0, 7.2] | 3.0 [2.7, 6.4] | 0.353 |
| MVA_2D (cm2) |  | 5.0 [1.3, 5.0] | 5.0 [1.7, 5.0] | 0.264 |
| MVA_PHT (cm2) |  | 5.0 [1.2, 5.0] | 5.0 [1.7, 5.0] | 0.701 |
| MR_VC (mm) |  | 0.0 [0.0, 0.0] | 0.0 [0.0, 0.0] | NA |
| tethering_hight (mm) |  | 6.0 [6.0, 6.0] | 6.0 [6.0, 6.0] | NA |
| TR (%) | normal | 278 ( 21.6) | 135 ( 20.1) | 0.134 |
|  | trivial | 214 (16.6) | 86 (12.8) |  |
|  | mild | 700 (54.5) | 395 (59.0) |  |
|  | moderate | 80 ( 6.2) | 48 ( 7.2) |  |
|  | severe | 14 ( 1.1) | 6 ( 0.9) |  |
| TV_TR_PG (mmHg) |  | 22.6 [0.0, 77.1] | 23.9 [0.0, 71.7] | 0.012 |
| TV_estimation_PAPs (mmHg) |  | 25.0 [7.0, 80.0] | 27.0 [14.0, 80.0] | 0.007 |
| PS (%) | normal | 1285 ( 99.9) | 666 ( 99.9) | 0.126 |
|  | mild | 1 ( 0.1) | 1 ( 0.1) |  |
| PR (%) | normal | 1158 ( 90.0) | 608 (90.7) | 0.079 |
|  | trivial | 28 ( 2.2) | 23 ( 3.4) |  |
|  | mild | 89 ( 6.9) | 34 ( 5.1) |  |
|  | moderate | 11 ( 0.9) | 5 ( 0.7) |  |
| PV_PR_PG (mmHg) |  | 6.0 [1.9, 13.0] | 6.00 [3.0, 13.0] | 0.622 |
| PV_estimate_LVEDP (mmHg) |  | 12.0 [5.0, 13.0] | 12.0 [6.0, 12.0] | 0.904 |
| seg_SUM |  | 17.0 [4.0, 59.0] | 17.0 [10.0, 51.0] | 0.75 |
| seg_Average |  | 1.0 [1.0, 3.5] | 1.0 [1.0, 3.0] | 0.842 |

S2 Table. Transthoracic echocardiography measurement.

Data are presented as median [min, max], (%).

TTE, transthoracic echocardiography; HR, heart rate; Ao, aorta; LA, left atrium; IVSth, interventricular septum thickness; LVPWth, thickness of the left ventricular posterior wall; LVDd, left ventricular diameter at the end diastole; LVDs, left ventricular diameter at the end systole; FS, fractional shortening; EDV, end-diastolic volume; ESV, end-systolic volume; SV, stroke volume; EF, ejection fraction; TMF, transmitral flow; DcT, deceleration time; PVF, pulmonary vein flow; IVS, intraventricular septum; Lat, lateral; IVC, inferior vena cava; LVOT, left ventricular outflow tract; PG, pressure gradient; CO, cardiac output; CI, cardiac index; RA, right atrium; RVDd, right ventricular diameter at end diastole; RVWth, thickness of the right ventricular posterior wall; AS, aortic stenosis; AR, aortic regurgitation; AV, aortic valve; AVA, aortic valve area; BSA, body surface area; PHT, pressure half time; VC, vena contracta; STJ, ST junction; MS, mitral stenosis; MR, mitral regurgitation; MV, mitral valve; MVA, mitral valve area; TR, tricuspid regurgitation; TV, tricuspid valve; PAP, pulmonary artery pressure; PS, pulmonary stenosis; PR, pulmonary regurgitation; LVEDP, left ventricular end-diastolic pressure
